# Zonal Rate Model for Axial and Radial Flow Membrane Chromatography. Part I: Knowledge Transfer Across Operating Conditions and Scales

**DOI:** 10.1002/bit.24771

**Published:** 2012-11-23

**Authors:** Pranay Ghosh, Kaveh Vahedipour, Min Lin, Jens H Vogel, Charles A Haynes, Eric von Lieres

**Affiliations:** 1 IBG1: Biotechnology, Forschungszentrum JülichWilhelm-Johnen-Strasse 1, 52425 Jülich, Germany; telephone: +49-2461-61-2168; fax: 49-2461-61-3870; 2 INM-4: Medical Imaging PhysicsForschungszentrum, Jülich, Germany; 3 Isolation and Purification Department, Global Biologics Development, Bayer HealthcareBerkeley, California; 4 Michael Smith Laboratories, University of British ColumbiaVancouver, British Columbia, Canada

**Keywords:** membrane chromatography, modeling, process analysis

## Abstract

The zonal rate model (ZRM) has previously been applied for analyzing the performance of axial flow membrane chromatography capsules by independently determining the impacts of flow and binding related non-idealities on measured breakthrough curves. In the present study, the ZRM is extended to radial flow configurations, which are commonly used at larger scales. The axial flow XT5 capsule and the radial flow XT140 capsule from Pall are rigorously analyzed under binding and non-binding conditions with bovine serum albumin (BSA) as test molecule. The binding data of this molecule is much better reproduced by the spreading model, which hypothesizes different binding orientations, than by the well-known Langmuir model. Moreover, a revised cleaning protocol with NaCl instead of NaOH and minimizing the storage time has been identified as most critical for quantitatively reproducing the measured breakthrough curves. The internal geometry of both capsules is visualized by magnetic resonance imaging (MRI). The flow in the external hold-up volumes of the XT140 capsule was found to be more homogeneous as in the previously studied XT5 capsule. An attempt for model-based scale-up was apparently impeded by irregular pleat structures in the used XT140 capsule, which might lead to local variations in the linear velocity through the membrane stack. However, the presented approach is universal and can be applied to different capsules. The ZRM is shown to potentially help save valuable material and time, as the experiments required for model calibration are much cheaper than the predicted large-scale experiment at binding conditions. Biotechnol. Bioeng. 2013; 110: 1129–1141. © 2012 Wiley Periodicals, Inc.

## Introduction

Packed bed chromatography is one of the most widely employed purification steps in biopharmaceutical industry. High resolution makes it an indispensable unit operation in the recovery of therapeutic proteins and recombinant drugs where purity is of utmost importance. In the recent past, an ever-growing market demand of drugs has led to the production of large batch volumes, which has put an enormous pressure on downstream processing for including higher throughput operations than conventional packed bed chromatography for rapid product isolation (Levine, 2002; Przybycien and Pujar, 2004). Membrane chromatography is a very attractive alternative due to many beneficial features (Endres et al., 2003; Ghosh, 2001; Klein, 2000; Przybycien and Pujar, 2004; Saxena et al., 2009; Teeters et al., 2003; Vogel et al., 2012), such as the potential of working at higher flow rates while maintaining binding capacities at comparable levels to packed bed chromatography. Membrane chromatography occupies smaller footprints because of the smaller size of membrane chromatography capsules as compared to packed bed chromatography columns with similar volume processing capacities (Zhou and Tressel, 2006). Membrane chromatography capsules are often disposable, which can offer additional advantages over packed bed chromatography, as cleaning steps are not required.

The performance differences between resin based and membrane chromatography are mainly caused by different mass transfer regimes (Klein, 2000; Sarfert and Etzel, 1997; Suen and Etzel, 1994). In packed bed chromatography a column is filled with porous beads whose inner surfaces are functionalized with specific adsorption sites. During the loading step, a feed stream that contains target molecules, for example proteins, and various impurities is passed through this column. The target molecules (and the strongly binding impurities) are bound to the adsorption sites. The solute molecules are transferred from the bulk fluid to the binding sites by the successive mechanisms of convection, external mass transfer through a stagnant boundary layer around the beads, pore diffusion within the bead pores, and finally by adsorption. In packed bed chromatography, the pore diffusion step is often rate limiting and can prevent rapid mass transfer to the binding sites.

Membranes have much larger pores and the binding sites are better accessible, because the mass transfer from the bulk fluid to the binding sites is predominantly through convection. The protein molecules are transferred across a thin film layer and immediately bound to the adsorption sites. There is no mass transfer limitation due to pore diffusion, and film diffusion can be neglected in many cases (Dimartino et al., 2011; Francis et al., 2011; Frey et al., 1992; Gerstner et al., 1992; Shiosaki et al., 1994). Due to smaller bed height, membrane chromatography has lower pressure drops and can consequently be operated at higher flow rates. These advantages have been successfully utilized for the industrial removal of trace impurities such as plasmid DNA, host cell proteins, and for virus clearance in polishing steps (Knudsen et al., 2001; Tennikov et al., 1998; Zhou et al., 2006). Membranes are particularly useful for the separation of large protein molecules (*M*_w_ > 250 kDa) where better access to the binding sites is essential (Endres et al., 2003). The potential of working at high flow rates also allows to process larger volumes and, hence, membrane chromatography can be employed for volume reduction before expensive unit operations, such as Protein A affinity steps.

Membrane chromatography capsules are available in different configurations on the market (Zhou et al., 2006). The flat sheet arrangement with axial flow, in which the membranes are stacked in multiple layers in a small capsule, is usually available for lab scale applications and meant to provide a convenient scale-down approach for designing specific separation problems. In typical capsules for large-scale purification with radial flow, the membranes are either spirally wound or pleated around a core. As membrane chromatography has diverse applications and is increasingly accepted in biopharmaceutical industry, accurate models for different configurations become important for rational process analysis and design.

In the present contribution the previously published ZRM is extended from axial to radial flow configurations and applied for describing breakthrough data of both configurations under binding and non-binding conditions. A simultaneous analysis and evaluation strategy across scales and conditions is presented, and the potential of the proposed modeling approach for model-assisted scale-up is evaluated.

## Theory

**Figure 1:**
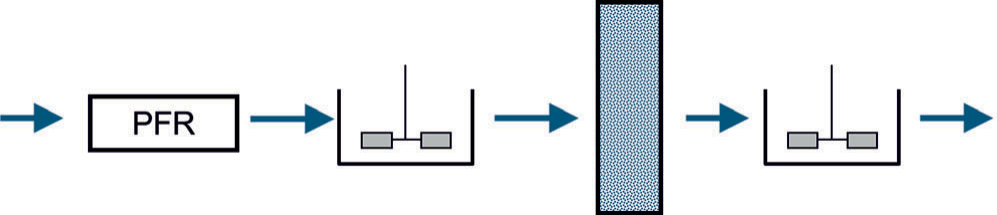
Traditional modeling of external hold-up volumes by a linear PFR and CSTR sequence Roper and Lightfoot model (Roper and Lightfoot, 1995).

Chromatography with stacked membranes has been modeled for many years (Boi, 2007; Boi et al., 2007; Roper and Lightfoot, 1995; Wang et al., 2008). In most studies the membranes are described in one spatial dimension along the axial coordinate, and external hold-up volumes are accounted for by a plug flow region (PFR) and one or two continuously stirred tank regions (CSTR) in series with the membrane (Fig. 1). Such models assume homogeneity over membrane cross-sections, which is practically hard to achieve in membrane capsules due to large length-to-width ratios. In fact, the external hold-up volumes of membrane chromatography capsules are typically in the same order of magnitude as the membrane volume, and therefore these volumes contribute significantly to solute dispersion apart from the membrane stacks. Thus, a linear sequence of interconnected PFR and CSTR is insufficient for describing the effect of these hold-up volumes (Montesinos-Cisneros et al., 2007; Sarfert and Etzel, 1997; Vicente et al., 2008).

**Figure 2:**
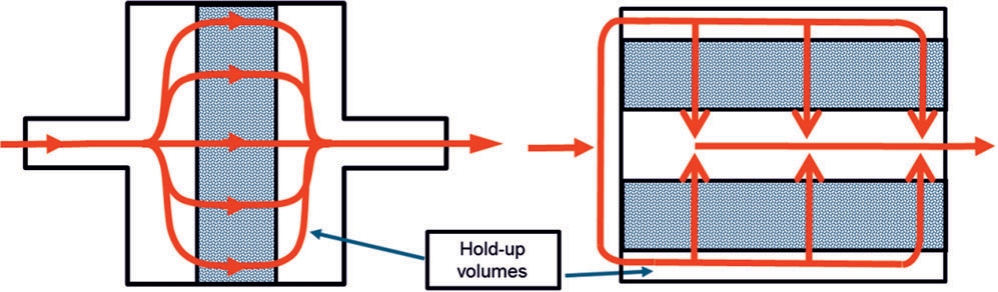
Different flow paths in axial and radial flow configurations.

In axial flow configurations several membrane sheets are stacked in capsules whose diameter is several orders of magnitude larger than the bed length. At such extreme length-to-diameter ratios the path lengths traversed by solute molecules that are passing through the outer radial region are much longer than the path lengths traversed by solute molecules that are passing through the central region (Ghosh and Wong, 2006) (Fig. 2). A similar situation is found in radial flow configurations where the feed stream is split into different fractions before reaching the membrane that is either spirally wound or pleated around a cylindrical core.

ZRM (Francis et al., 2011, 2012) has been developed for quantitatively analyzing the impact of radially inhomogeneous flow distributions on measured chromatograms under non-binding and binding conditions for axial flow configurations. The concept of the ZRM is to virtually partition the hold-up volumes before and behind the membrane as well as the membrane stack itself into different zones. Each zone is considered homogenous but of different size and subjected to different boundary conditions depending on its relative position in the given arrangement. The interconnected virtual zones for the hold-up volumes are modeled as a network of CSTRs. The virtual zones of the membrane stack are described by several instances of a conventional membrane chromatography model. The inflows of these identical instances are connected to different CSTRs in the network and, hence, subject to different boundary conditions. A PFR is connected in series with the CSTR and membrane model network in order to account for possible time lags that are not associated with system dispersion. The ZRM is a semi-empirical approach for independently quantifying the impacts of binding kinetics and internal flow distributions in membrane chromatography units without knowing the internal capsule geometry.

In this work the ZRM is further extended to radial flow configurations. The radial flow configuration has a different flow pattern as the axial flow configuration and therefore requires a different set-up and interconnection of virtual zones. In the following sections the differences in setting up the ZRM for axial and radial flow configurations are highlighted.

### Zonal Rate Model for Axial Flow Configuration

**Figure 3:**
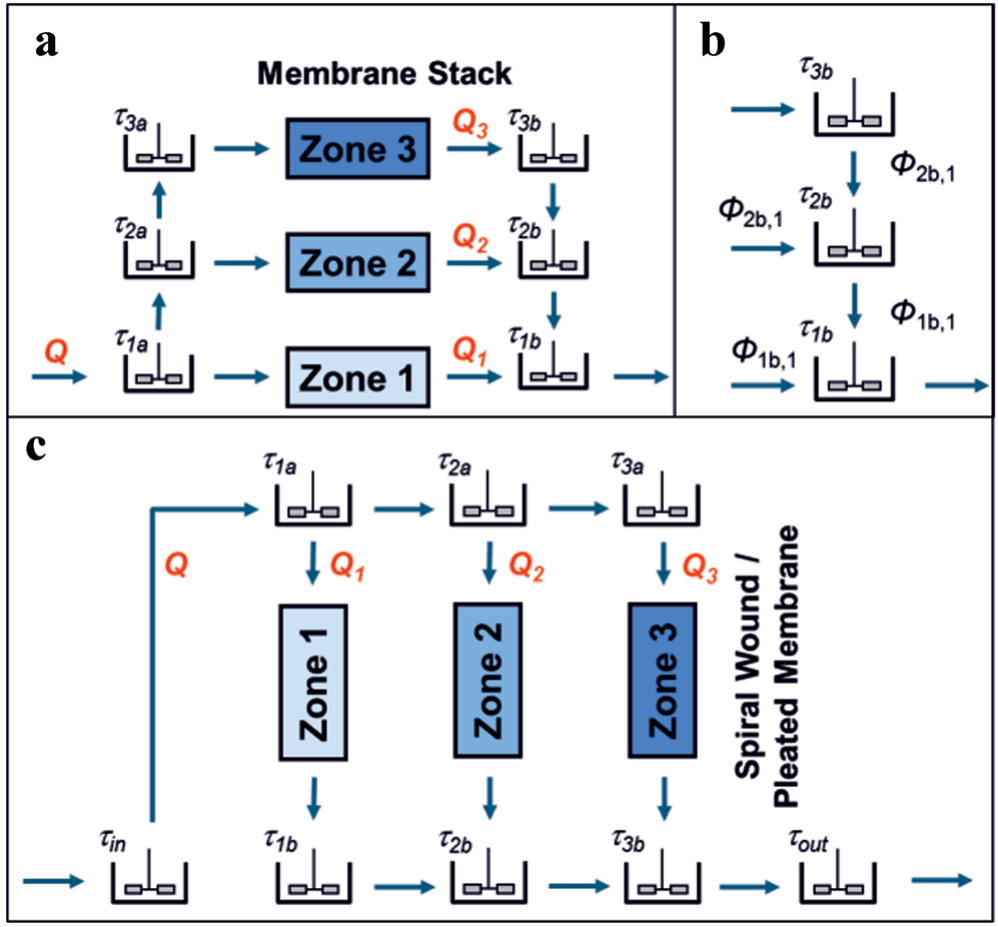
**a**: Virtual partitioning of hold-up volumes and of the membrane for an axial flow configuration with three membrane zones, (**b**) flow fractions for tanks downstream of the membrane, and (**c**) virtual partitioning of hold-up volumes and of the membrane for a radial flow configuration with three membrane zones.

A detailed description of the ZRM for different axial flow configurations has been published earlier (Francis et al., 2011, 2012; von Lieres et al., 2010). Figure 3a illustrates the modeling approach for a configuration with three virtual zones for the hold-up volumes before and behind the membrane stack as well as for the membrane stack itself. Solute molecules that cross the outermost zone of the membrane stack are sequentially passed through tanks 1a, 2a, and 3a; the respective membrane zone, and tanks 3b, 2b, and 1b. Solute molecules that cross the central zone of the membrane stack are only passed through tank 1a, the respective membrane zone, and tank 1b. Average residence times of solute molecules that pass the capsule along different paths are calculated as the sum of residence times of the passed tanks and of a PFR in series with the CSTR network plus the residence time in the respective membrane zone.

### Zonal Rate Model for Radial Flow Configuration

Different capsules with radial flow configuration are available on the market. The ZRM has been developed as a flexible tool for individually studying the effects of inhomogeneous flow and of solute molecule binding in different design types of membrane chromatography capsules. The radial flow configuration of the ZRM is derived for capsules in which the solute molecules pass through the membrane from the periphery to the center of the capsule (Fig. 2), but a similar configuration can be derived for the opposite flow direction. As illustrated in Figure 3c, the feed flow is redirected and distributed over an outer peripheral channel, then perpendicularly crosses the membrane and is collected in a central cylindrical channel before exiting the capsule. Extra tanks are incorporated at the inlet and outlet of the capsule in order to account for the hold-up volumes of the distributor and collector regions. The Roper and Lightfoot model (Fig. 1) is also a special case of the radial flow configuration with only one membrane zone. However, this model must be set-up with non-identical CSTR regions upstream and downstream of the membrane in order to account for the inherent asymmetry of radial flow capsules.

The solute molecules can be sequentially passed through the inlet zone and zone 1a, the inlet zone, zone 1a and zone 2a, or through the inlet zone, zones 1a, 2a, and zone 3a before reaching the outer side of the membrane. The fractions of the overall volumetric flow that pass through the individual membrane zones are given by *Q*_1_, *Q*_2_, and *Q*_3_. In contrast to the axial flow configuration, all solute molecules pass though the same number of tanks, independent of their individual flow paths, but the residence times of the central tanks are significantly smaller than those of the corresponding tanks in the periphery. Hence, the average residence time, which is the sum of the residence times of the passed tanks plus of the crossed membrane zone, does depend on the flow path. The individual PFR and CSTR equations are set-up identical as for the axial flow configuration, but differently interconnected with each other and with the model instances for the membrane zones.

### Transport Equations

Each virtual zone of the membrane stack is described by an instance of the same mass-balance equation (Eq. 1).


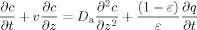
(1)

In Equation (1), *c* and *q* are the solute concentrations in the mobile and stationary phases, respectively, *v* is the flow velocity, *D*_a_ is the dispersion coefficient, and ∂*q*/∂*t* denotes the rate of adsorption or desorption of solute molecules to or from the membrane surface. The stationary phase concentration is accounted for per unit volume of solid membrane, and the dispersion coefficient has been shown earlier to contribute negligibly to the total system dispersion (Francis et al., 2011, 2012). The binding of solute molecules will be discussed in Binding Kinetics Section. A PFR is added in series with the CSTR network for modeling the time-lag in the breakthrough curve that is caused in the system dead volume and not associated with system (Eq. 2).


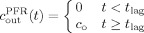
(2)

In Equation (2), *t*_log_ is the ratio of the PFR volume to the volumetric feed flow rate, *V*_PFR_/*Q*. Several CSTR models are connected for describing solute dispersion in the hold-up volumes upstream and downstream of the membrane stack. Equation (3) is used for tanks with one feed stream, where 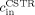 and *c*^CSTR^ are the solute concentrations at the tank inlet and outlet, respectively, *τ* = *V*/*Q* is the average residence time of solute molecules, *V* is the tank volume, and *Q* is the volumetric flow rate.


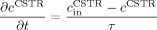
(3)

Tanks with two or more feed stream, that are required downstream of the membrane, are described by Equation (4), where *j* is the number of tank inlets.


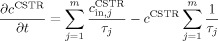
(4)

The ZRM also includes a set of flow fractions, *Φ*_*k*_, which define the fraction of the total volumetric flow, *Q* that passes through each of the membrane zones. Figure 3b illustrates this for an example with three membrane zones. Let *Φ*_1_, *Φ*_2_, and *Φ*_3_ denote the fractions of the total volumetric flow that pass through the membrane zones. Due to mass conservation, the sum of these flow fractions must equal one (*Φ*_1_ + *Φ*_2_ + *Φ*_3_ = 1). Moreover, let *Φ*_1b,1_ and *Φ*_1b,2_ denote the flow fractions of the two inlets of the first tank, and *Φ*_2b,1_ and *Φ*_2b,2_ the same for the second tank. The sum of these flow fractions of individual tanks also equals one (*Φ*_1b,1_ + *Φ*_1b,2_ = 1 and *Φ*_2b,1_ + *Φ*_2b,2_ = 1). In the given scenario, Equation (4) is set up for each of these tanks with residence time 1/*τ*_1b,1_ + 1/*τ*_1b,2_ = 1/*τ*_1b_ and 1/*τ*_2b,1_ + 1/*τ*_2b,2_ = 1/*τ*_2b_. The corresponding flow fractions are *Φ*_1b,*i*_ = *τ*_1b_/*τ*_1b,*i*_ and *Φ*_2b,*i*_ = *τ*_2b_/*τ*_2b,*i*_ for 1 ≤ *i* ≤ 2. The flow fractions through the membrane zones are then determined by *Φ*_1_ = *Φ*_1b,1_, *Φ*_2_ = *Φ*_1b,2_*Φ*_2b,1,_ and *Φ*_3_ = *Φ*_1b,2_*Φ*_2b,2_. Similar relations can be analogously derived for more complex networks.

### Binding Kinetics

Several models have been published for describing the binding of solute molecules to functionalized surfaces. Protein adsorption is a complex process, and the variety of involved physical mechanisms can hardly be included in binding isotherms that are used for practical applications. The rather simple Langmuir kinetic (Eq. 5) is often applied for modeling protein adsorption and desorption to and from ion-exchange membranes (Gebauer et al., 1997; Suen and Etzel, 1994).


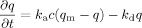
(5)

In Equation (5), *k*_a_ and *k*_d_ are the adsorption and desorption rate constants and *q*_m_ is the maximum binding capacity. The Langmuir model assumes single-component interaction with one type of binding sites of solute molecules that do not interact with each other.

Several more complex modeling approaches have been developed for describing adsorption and desorption of proteins and other biomolecules. For instance, large molecules can cause steric hindrance and pore blocking can entirely prevent the adsorption of further molecules. The adsorbate molecules can also interact with each other and form dimers and other aggregates, and the aggregate species interact differently with the adsorption sites. Furthermore, adsorbent materials can provide more than one binding mechanism. It can also be assumed that since adsorption is an energetically driven process, the adsorbate molecules can undergo conformation or orientation changes in order to minimize the free energy during adsorption. The latter is described by the spreading model (Clark et al., 2007), which is used in the present study for overcoming the limitations of the Langmuir model.

**Figure 4:**
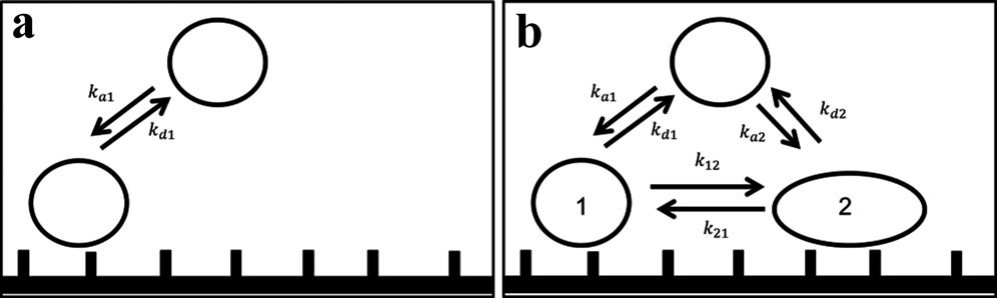
Adsorption schemes in the (**a**) Langmuir and (**b**) spreading models.

Etzel and coworkers have successfully applied the spreading model for describing the asymmetric breakthrough behavior of protein loading on membrane surfaces (Yang and Etzel, 2003). A recent study of the Hubbuch group provides evidence for the existence of different binding orientations of lysozyme on ion-exchange beads (Florian Dismer, 2007). In a comparative study of different binding models in membrane chromatography (Francis et al., 2011), the spreading model has been found to be most suitable for quantitatively describing the adsorption of ovalbumin on a modified polyethersulfone (PES) membrane with cationic groups. The shape of BSA, which is used in the present study, has been reported as a cigar shaped ellipsoid (7 nm × 4 nm× 4 nm) (Kim, 2002). Hence BSA can physically absorb to the membrane surface in two different orientations, at the end or sideways. The most general form of the spreading model for two orientations of the molecules is given by Equations (6)–(8). The protein molecules can be bound and released in both orientations but with different rate constants and also change their orientation in the bound state (Fig. 4). The total amount of bound protein is given by the sum of both orientations. In contrast to the bi-Langmuir model, both orientations compete for the same binding sites.


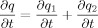
(6)


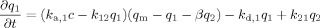
(7)


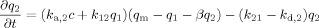
(8)

In Equations (6)–(8), *q*_1_ and *q*_2_ represent the concentrations of bound protein in orientation 1 and 2, respectively, *β* is the ratio of the sorbent surface area occupied by a bound protein in state 2 relative to that in state 1, the binding rate constants *k*_a,1_, *k*_d,1_, *k*_a,2,_ and *k*_d,2_ are defined in analogy to the Langmuir model, and the constants *k*_12_ and *k*_21_ describe the rates of the orientation change.

## Materials and Methods

Bovine serum albumin (BSA) (A 7638, Sigma–Aldrich Corp., St. Louis, MO) was used in breakthrough experiments at a concentration of 1 g/L and flow rates of 12 CV/min for both the axial and radial flow membrane chromatography capsules. The protein was dissolved in 25 mM Tris buffer at pH 8.0 (Sigma–Aldrich) for the loading step. Loading was followed by a washing step with 25 mM Tris buffer at pH 8.0. Then, 1 M NaCl in 25 mM Tris buffer pH 8.0 was used to elute the bound BSA from the membranes. The units were cleaned with 1 N NaOH as specified by the manufacturer after each run. In a revised protocol the cleaning step was performed with 1 M NaCl instead of 1 N NaOH.

Mustang Q XT5 anion-exchange membrane chromatography capsules (axial flow) and Mustang Q XT140 anion-exchange membrane chromatography capsules (radial flow) were purchased from Pall, Inc. (East Hills, NY). Both capsules contain modified hydrophilic polyethersulfone (PES) membranes whose surfaces are coated with an irreversibly cross-linked polymer that contains pendant Q groups. In the XT5 capsules 15 layers of flat membrane sheets are stacked with a bed height of 2.20 mm and a frontal area of 22.06 cm^2^. In the XT140 capsules membrane pleats are arranged in a radial flow configuration with a pleat length of 7.6 cm and a pleat width of 2.8 cm. The effective bed height of the membrane stacks in the XT140 capsule is also 2.20 mm. The pore size and porosity ε of the membrane are 0.8 µm and 0.70 ± 0.05, respectively (manufacturer data).

The total membrane volume in the XT5 capsule is 5 mL, and the hold-up volumes upstream and downstream of the membrane stack are 3.21 mL each. The XT5 capsule was attached to a ÄKTAexplorer system that was controlled by the Unicorn software (GE Healthcare, Uppsala, Sweden). The total membrane volume in the XT140 capsule is 140 mL, and the hold-up volumes upstream and downstream of the pleated membrane are 105 and 45 mL, respectively. The XT140 capsule was attached to a ÄKTAprocess system that was controlled by the Unicorn software (GE Healthcare). An experimental 9.4 T magnetic resonance tomography device (MRT) was used for visualizing the internal geometry of both capsules.

The model equations for the virtual membrane zones, continuously stirred tank and PFR were coupled together, resulting in a large set of differential equations. The space derivatives in the membrane model instances were first discretized with a finite difference method. The MATLAB solver *ode15s* was then used for integrating the forward problem for given parameter values over time. A highly efficient computational algorithm has been implemented that solves typical set-ups of the ZRM in 2–3 s on a standard desktop computer with 2 GHz. The inverse problem, that is estimating unknown model parameters from breakthrough data, is iteratively solved using the MATLAB optimizer *lsqnonlin*. A multi-start strategy is employed for avoiding local optima in the parameter space.

## Results and Discussion

The Pall XT5 and XT140 capsules were studied by analyzing breakthrough curves that have been measured under binding and non-binding conditions. A flow rate of 12 CV/min was chosen for studying the capsules under industrially relevant conditions. The effects of different flow rates in the axial flow configuration have been described in a previous publication (Francis et al., 2011).

### Axial Flow Configuration at Non-Binding Conditions

Breakthrough experiments performed under non-binding conditions provide insights into solute dispersion within the studied membrane chromatography capsules. Non-binding conditions are obtained by adding 1 M NaCl to the protein solution. The analysis of non-binding data is crucial for quantifying the effect of system non-idealities that are caused by inhomogeneous flow separately from non-ideal binding mechanisms.

**Figure 5:**
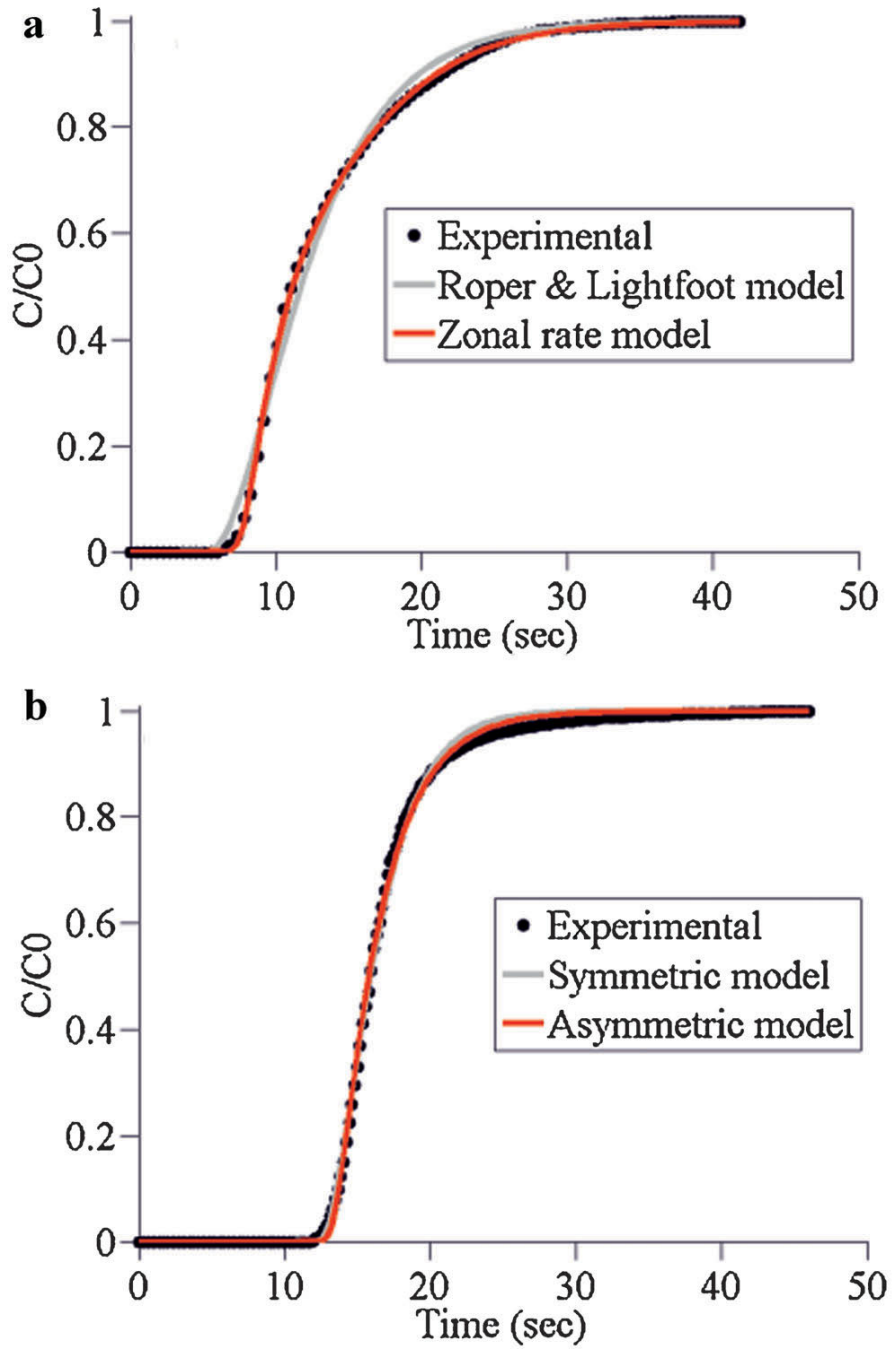
Measured breakthrough curve of the axial flow XT5 capsule under non-binding conditions. **a**: Best fit of the symmetric Roper and Lightfoot model and of the symmetric zonal rate model (ZRM) with two membrane zones for XT5 capsule, (**b**): best fit of the symmetric and asymmetric ZRM with one membrane zone (Roper and Lightfoot model) for XT140 capsule.

Breakthrough experiments under non-binding conditions provide information on the sum of the hold-up volumes in the Äkta system and in the studied chromatography capsule. The chromatography system was primed with load material up to the column switch valve in order to effectively remove the impact of the hold-up volumes before that point, as the corresponding system components, that is pumps, mixer and tubing, are already filled with protein solution when the valve is switched from bypass to the capsule. Consequently, the dispersion in the measured breakthrough curve (Fig. 5a) is caused only by the chromatography capsule and by the hold-up volumes behind that capsule that is the tubing and the detection chamber. However, the hold-up volumes behind the capsule sum up to only 18 µL and, hence, their contribution to system dispersion can be neglected.

After an initial lag of 6 s the signal rapidly increases to half of the inlet concentration in 4 s but then gradually flattens out and takes approximately 20 more seconds for reaching the full inlet concentration. The observed tailing is far from the ideal system response but rather typical for membrane chromatography units with extreme length-to-with ratios, even though the dispersion of solute molecules on their short path through the membrane stack itself can often be neglected. Francis et al.(2011, 2012) have studied the same capsule and shown that the dispersion coefficient in the model for the membrane stack can be replaced by the molecular diffusion coefficient when the hold-up volumes are properly described. The same approach is followed in the present study. The membrane stack in the analyzed XT5 capsule has a volume of 5 mL while the hold-up volumes on the either side of the stack are 3.21 m. The membrane stack has a porosity of 0.7 (Pall XT5, which implies that the capsule contains only 1.5 mL of solid membrane. Hence, the total hold-up volume of the capsule actually exceeds the membrane volume.

As a reference, the Roper and Lightfoot model with a linear sequence of PFR and two CSTRs, one before and one after the membrane stack, is fitted to the measured breakthrough curve (see Fig. 5a). The residence times for the tanks on either side of the membrane stack are chosen identical in order to account for the respective symmetry of the studied XT5 capsule. Hence, two parameters of the Roper and Light foot model are estimated from measurement data, namely the residence times of the PFR and of the CSTRs (see Table I). The Roper and Lightfoot model can only coarsely approximate the measured breakthrough curve. The simulated curve not only shows a delay of the initial breakthrough but also a reduced tailing as compared to the measurement data.

**Table I:** Hold-up volumes and volumetric flow fractions as determined by fitting the zonal rate model (ZRM) with one membrane zone (Roper and Lightfoot model) and with two membrane zones to a non-binding breakthrough curve of the axial flow XT5 capsule (*V*_PFR_ = *Qt*_PFR_, *V*_inner_ = *Q*τ_1_, *V*_outer_ = *QΦ*_2_τ_2_)

Parameter	One membrane zone (mL)	Two membrane zones (mL)
*V*_PFR_	2.22	3.91
*V*_inner_	3.64	1.24
*V*_outer_	—	1.69
*Φ*_2_	—	0.43

The ZRM was set-up with differently many membrane zones, also assuming symmetry of the tanks before and behind the membrane stack. Each of these configurations was fitted to the data in order to determine the minimal number of membrane zones that is required for quantitatively reproducing the measured breakthrough curve. In full agreement with previous results for the same capsule (Francis et al., 2011), a set-up with two membrane zones was found to be optimal. This set-up has four parameters, namely the PFR volume, the different volumes of two CSTRs for describing the hold-up zones before the membrane stack (the hold-up zones behind the stack are symmetrically modeled), and the volumetric flow fraction between the two membrane zones. The values of these parameters (see Table I) are completely determined by the internal geometry of the studied capsule. Although the inner tank has a smaller volume, 57% of the volumetric flow passes through the central region. The determined total hold-up volume of the capsule of 2.91 mL is reasonably close to the manufacturer specification of 3.21 mL. The ZRM is a semi-empirical approach that is based on the physical geometry, but a good reproduction of the experimental data is preferred over a perfect match of the physical volumes. A restriction of the total hold-up volume in the ZRM would remove one degree of freedom from the parameter estimation. However, the entire breakthrough curve would be shifted to the right, and this effect could not be compensated for by taking more zones, as the total hold-up volume is proportional to the area over the breakthrough curve. Computational fluid dynamics allows for a more stringent description of the internal capsule geometry, however, this would require much higher modeling and computing efforts and goes beyond the scope of the present study.

### Radial Flow Configuration at Non-Binding Conditions

Non-binding breakthrough data of the radial flow XT140 capsule were also analyzed with different set-ups of the ZRM. Similar to the XT5 capsule, the impact of the system hold-up volumes is reduced by priming the chromatography system with load material up to the column-switching valve. The remaining hold-up volumes in the tubing before and behind the XT140 capsule and in the detection chamber add up to 100 mL and cannot be completely neglected. However, the analysis in the following paragraph indicates that these external hold-up volumes mainly contribute to the PFR volume and not to the CSTR volumes in the ZRM. The resulting shift of the breakthrough curve does not affect the observed performance of the XT140 capsule.

Although the internal geometry of the XT140 capsule is more complex than of the XT5 capsule, a ZRM set-up with just one membrane zone was found to be sufficient for quantitatively reproducing breakthrough curves at the studied flow rate. However, an asymmetric model with unequal volumes before and behind the membrane is required (see Fig. 5b). A second membrane zone increases the number of regression parameters, but does not significantly improve the fit (data not shown). Hence, the asymmetric model with one membrane zone is used in the following sections. The sufficiency of one membrane zone indicates that the flow is distributed more homogeneously in the XT140 capsule than in the XT5 capsule. The substantially different tank volumes upstream and downstream of the membrane stack (see Table II) reflect the fact that, in contrast to the XT5 capsule, the peripheral distribution region with 105 mL and central collection region with 45 mL in the XT140 capsule are actually not symmetric. The fitted CSTR volumes in the ZRM are smaller, which indicates that a fraction of the rather complex shaped hold-up volumes within the XT140 capsule can be modeled as a PFR. The external hold-up volumes in the tubing and in the detector chamber are much more streamlined and will, consequently, predominantly contribute to the PFR volume in the ZRM, which does not contribute to system dispersion.

**Table II:** Hold-up volumes as determined by fitting the symmetric and the asymmetric ZRM with one membrane zone (Roper and Lightfoot model) to a non-binding breakthrough curve of the radial flow XT140 capsule (*V*_PFR_ = *Qt*_PFR_, *V*_upstream_ = *Qτ*_1_, *V*_downstream_ = *Qτ*_2_)

Parameter	Symmetric model (mL)	Asymmetric model
*V*_PFR_	259	269.93
*V*_upstream_	55.16	82.88
*V*_downstream_	Same as *V*_upstream_	19.32

### Axial Flow Configuration at Binding Conditions

In the previous two sections the impacts of flow non-idealities within the studied membrane chromatography capsules on experimentally measured breakthrough curves were individually analyzed under non-binding conditions. The internal geometry of the studied capsules was characterized by parameter values that represent residence times in virtual zones and flow fractions between these zones. These parameters are now fixed in order to independently analyze the impact of protein binding on the observed breakthrough curves with the Langmuir and spreading models.

**Figure 6:**
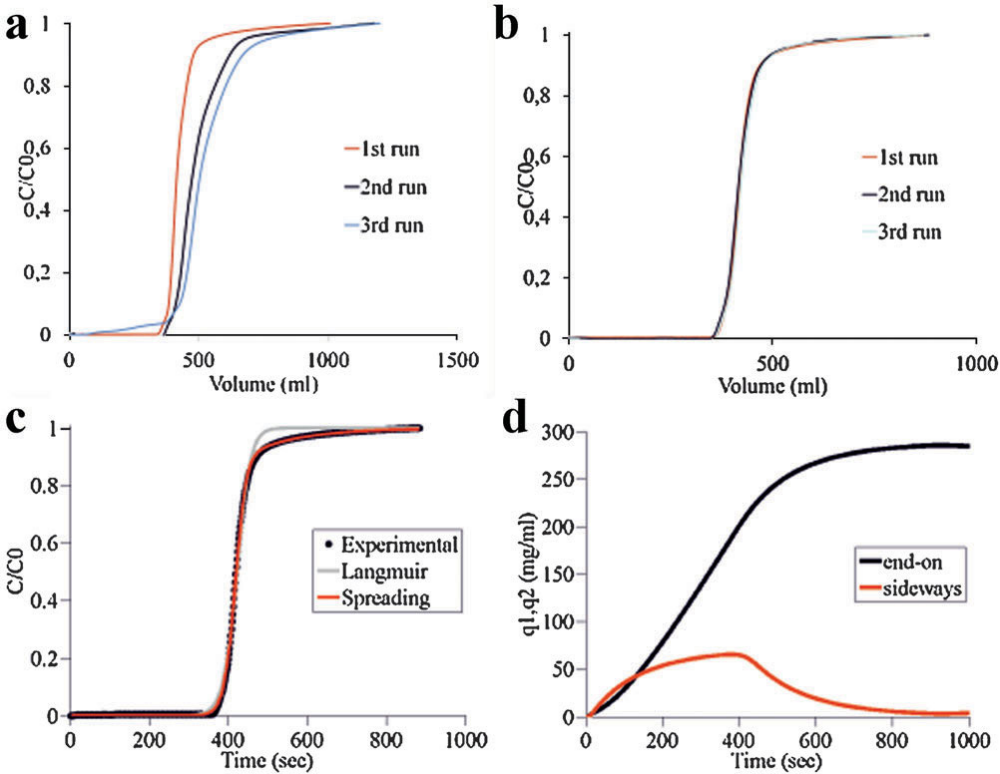
Measured breakthrough curve of the axial flow XT5 capsule under binding conditions. **a**: Using 1 N NaOH for cleaning after each run, (**b**): using 1 M NaCl for cleaning after each run, (**c**): best fit of the ZRM combined with the Langmuir binding model and the spreading model, and (**d**): simulated concentrations of bound molecules in the end-on orientation (q1: red line) and in the sideways orientation (q2: black line) during the loading process over time.

**Figure 7:**
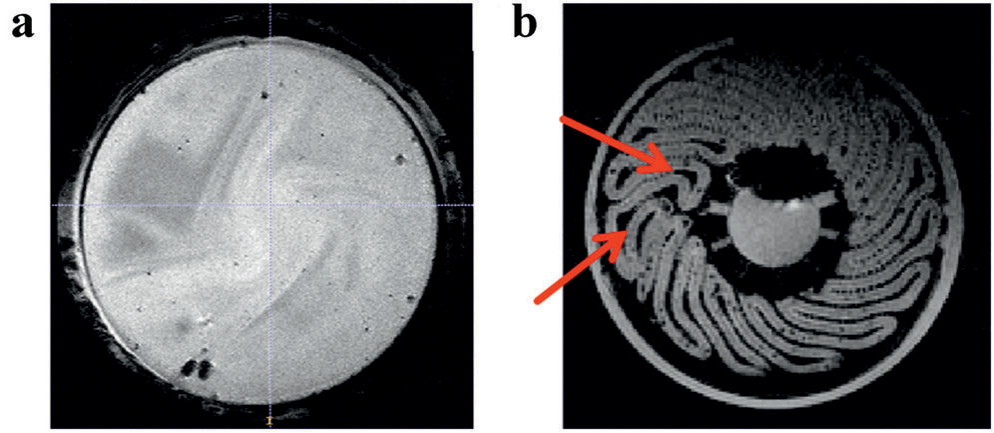
**a**: Cross-sectional MRI scan through the center of the membrane stack of an axial flow XT5 capsule that has been cleaned using 1 N NaOH, (**b)**: Cross-sectional MRI scan of the XT140 capsule. The membrane pleats are clearly visible in gray, due to their water content.

In the first binding experiments, the capsule was cleaned using 1 N NaOH after each run as specified by manufacturer. The cleaning step was followed by a regeneration step with 1 M NaCl. However, this protocol resulted in a very poor reproducibility (see Fig. 6a). Huge variations are observed for subsequent runs that were performed with the same capsule and under the same conditions. The exact reasons for the observed variations between measured breakthrough curves under the same conditions cannot be cogently explained, which poses a challenge in developing a coherent model. A possible explanation could be based on the fact that individual sheets of the membrane stack can slightly move within the XT5 capsule, and that swelling and de-swelling during treatment with NaOH might cause changes in the membrane position and shape. An MRI image of the membrane capsule after repeated cleaning with 1 N NaOH (see Fig. 7a) shows an uneven membrane surface with several wedges that could potentially cause preferential flow. The MRI investigation of membrane chromatography capsules will be continued in a separate study.

This study was continued with a fresh capsule and with a revised cleaning protocol in which 20 CV of 1 M NaCl were passed through the capsule after each completed cycle of load, wash and elution. Moreover, the time between two experiments was minimized by performing all runs immediately one after another. The revised protocol resulted in a much improved reproducibility of the breakthrough curve shapes (see Fig. 6b).

Rapid execution of the experiments was observed to be crucial, as the breakthrough curves were shifted to the right after the membrane was stored in the cleaning buffer (1 N NaOH) for several hours (data not shown). Similar shifts of the breakthrough curves are observed for the radial flow XT140 capsule that contains the same type of membrane (Radial Flow Configuration at Binding Conditions Section). These shifts, which are also observed in industrial applications of the same capsules, indicate that the overall binding capacity increases with storage time in the cleaning buffer. The cause is unclear, but the capacity would increase if the polyethylene sulfone (PES) backbone of the membrane had an inherent binding capacity for BSA and if the storage in NaCl would expose more of this backbone to the protein. Alternatively, more of the Q ligands could be exposed after storage in the cleaning buffer.

The measured breakthrough curves in Figure 6b are asymmetric and show a sharp increase from the initial breakthrough point to ca. 90 percent of the inlet concentration, which is followed by a very slow rise towards 100% of the inlet concentration. Without further analysis, the experimental data does not reveal the origin of the observed tailing. The tailing could be exclusively caused by non-ideal flow in the hold-up volumes, but the binding process can also influence the breakthrough curve in a non-ideal way. Hence, a model-based data analysis is proposed for separately quantifying the impact of flow non-idealities and binding non-idealities. The ZRM was combined with the Langmuir model and the spreading model for analyzing the binding data.

The complexity of the spreading model was reduced by assuming that BSA cannot directly adsorb or desorb from or to the sideways orientation. The resulting model reproduces the measurements equally well (data not shown) with a lower number of regressed parameters and is hence preferred in order to avoid over-parameterization and over-fitting.

Figure 6c shows the best fit of the ZRM with the Langmuir model, and the estimated parameters are summarized in Table III. The Langmuir model can reproduce the initial breakthrough but not the tailing. The spreading model reproduces the entire breakthrough curve much better than the Langmuir model (see Fig. 6c), even with neglected adsorption and desorption of the second bound state. The spreading model involves six parameters, *k*_a1_, *k*_d1_, *k*_12_, *k*_21_, *q*_m,_ and *β*, that are also estimated from binding breakthrough data (see Table IV).

**Table III:** Parameters of the Langmuir model for the axial flow configuration as determined by fitting the ZRM to a binding breakthrough curve of the axial flow XT5 capsule

Parameter	Value
*k*_a_ (L/(g s))	6.4 × 10^−2^
*k*_d_ (L/s)	6 × 10^−3^
*q*_m_ (g/L)	284.04

**Table IV:** Parameters of the spreading model for the axial flow configuration as determined by fitting the ZRM to a binding breakthrough curve of the axial flow XT5 capsule

Parameter	Value
*k*_a1_ (L/(g s))	8.08 × 10^−2^
*k*_d1_ (L/s)	1.06 × 10^−5^
*k*_12_ L/(g s))	7.37 × 10^−4^
*k*_21_ (L/s)	9.41 × 10^−3^
*q*_m_ (g/L)	289.003
*β*	1.144

The spreading factor *β* is larger than one and, hence, the molecules that are bound in the first orientation require less space as in the second orientation. This indicates that the first orientation is with one end towards the surface, whereas the second bound state is in a sideways orientation. However, the fact that the spreading model fits the experimental data very well cannot be taken as final proof for the underlying hypothesis of different binding orientations, and conformational changes of the bound molecule might also be involved.

The initial adsorption rate of solute molecules to the unsaturated membrane in the end-on orientation is 1/(*k*_a1_ × *q*_m_) = 0.042 s and the reorientation rate to the sideways orientation is 1/(*k*_12_ × *q*_m_) = 4.69 s. Both rates are quite fast, but the desorption rate is 1/*k*_d1_ = 157 min, which indicates almost irreversible binding under the observed conditions with an overall loading time of 15 min. However, the reorientation rate from the sideways to the end-on orientation is 1/*k*_21_ = 106 s and, consequently, both directions of the reorientation process are relevant during the loading process. Figure 6d shows the simulated amounts of bound molecules in both orientations over time. The BSA molecules are first bound in end-on orientation but rapidly transferred to the sideways orientation, which requires more space. Hence the surface is quickly saturated within 20 s. Then bound molecules in sideways orientation are more slowly transferred back to the end-on orientation, making room for further binding in end-on state. More than 15 min are required for reaching the complete equilibrium between both bound states. These two phases can also be seen in the measured breakthrough curves. The first phase corresponds with the initial sharp increase and the second phase with the long tail of the experimental curve. The maximum in the sideways orientation curve occurs at the inflection point in the breakthrough curve at 420 s where approximately 90% of the inlet concentration is reached (compare with Fig. 6b).

### Radial Flow Configuration at Binding Conditions

**Figure 8:**
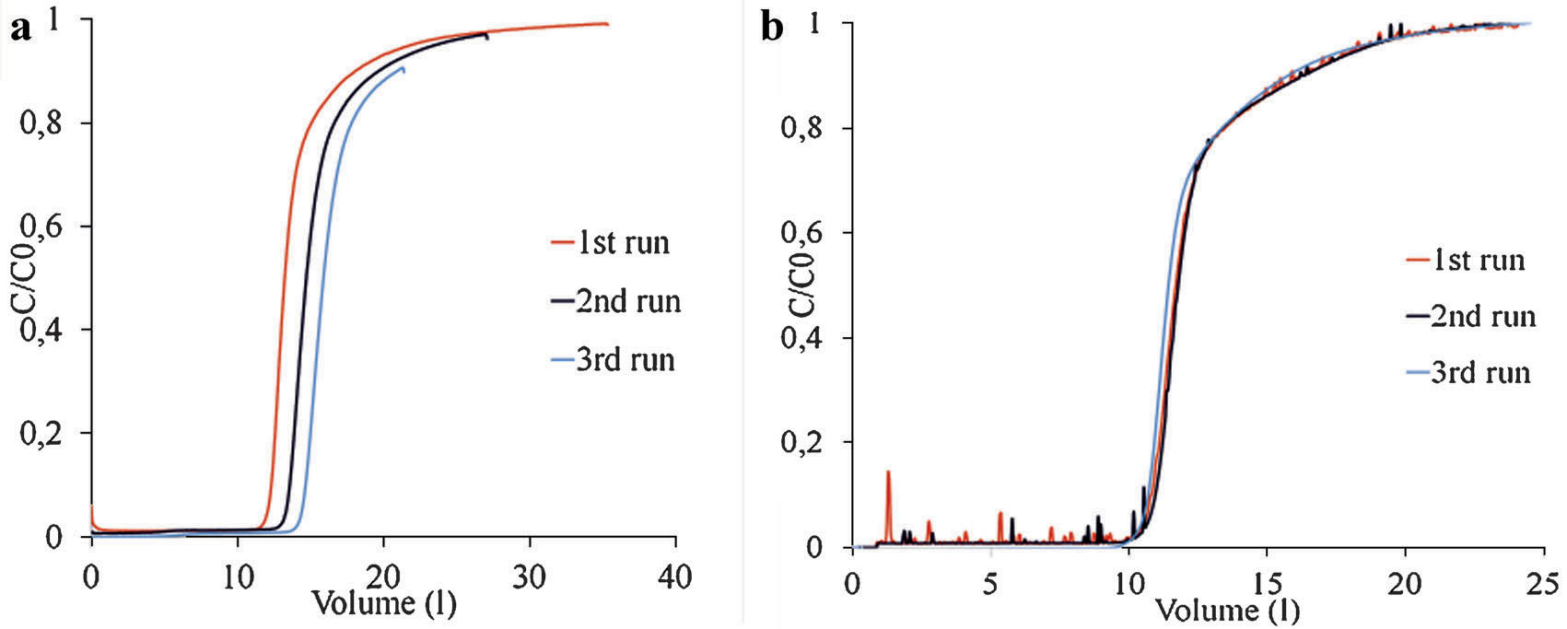
Measured breakthrough curve of the axial flow XT140 capsule under binding conditions. **a**: Using 1 N NaOH and (**b**): using 1 M NaCl for cleaning after each run.

With the manufacturer protocol for cleaning, the slopes of the measured breakthrough curves of the radial flow XT140 capsule were found to be better reproducible as compared to the XT5 capsule (see Fig. 8a). This might be due to the fact that in the XT140 capsule the membrane is not stacked but tightly arranged in fixed pleats, which effectively prevent position and shape changes. However, the breakthrough curves are also shifted to the right with increasing cycle numbers. Hence, the XT140 experiments were also performed with a fresh capsule and the revised cleaning protocol using 1 M NaCl instead of 1 N NaOH. The resulting breakthrough curves are not shifted but have similar shapes as compared to the original cleaning protocol (see Fig. 8b). The breakthrough curve in Figure 8b shows a sharply increasing section after the initial breakthrough point at 380 s in which 70% of the inlet concentration is reached within 420 s. The curve then gradually flattens out and reaches the full inlet concentration after approximately 850 more seconds.

**Figure 9:**
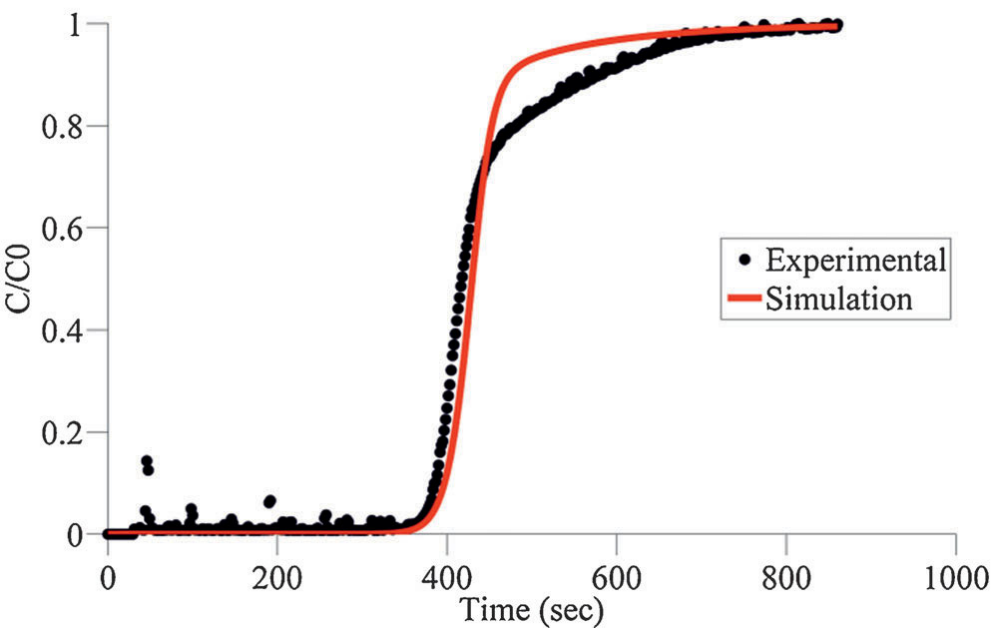
Predicted and measured breakthrough curve of the axial flow XT140 capsule under binding conditions. The asymmetric ZRM with one membrane zone was solved with the flow related parameters from Table III and the binding related parameters from Table V.

In Radial Flow Configuration at Non-Binding Conditions Section, the flow non-idealities in the XT140 capsule were described by an asymmetric ZRM with one membrane zone, and in Radial Flow Configuration at Binding Conditions Section, the kinetic parameters of the spreading model were determined independently from the flow configuration. Hence, the flow related parameters of the XT140 capsule (Table II) could be combined with the binding related parameters (Table IV) that have been determined for the same membrane type in the XT5 capsule. With this information, the ZRM can be applied for predicting breakthrough curves of the XT140 capsule under binding conditions. The result of this model-based prediction is compared to the corresponding measurement data in Figure 9. The simulated breakthrough curve closely matches the breakthrough point and the initial slope of the measured data. The model also correctly predicts the flattening of the breakthrough curve after 420 s, however, the predicted tail starts at 90% of the inlet concentration whereas the measured tail starts at 70% of the inlet concentration.

**Figure 10:**
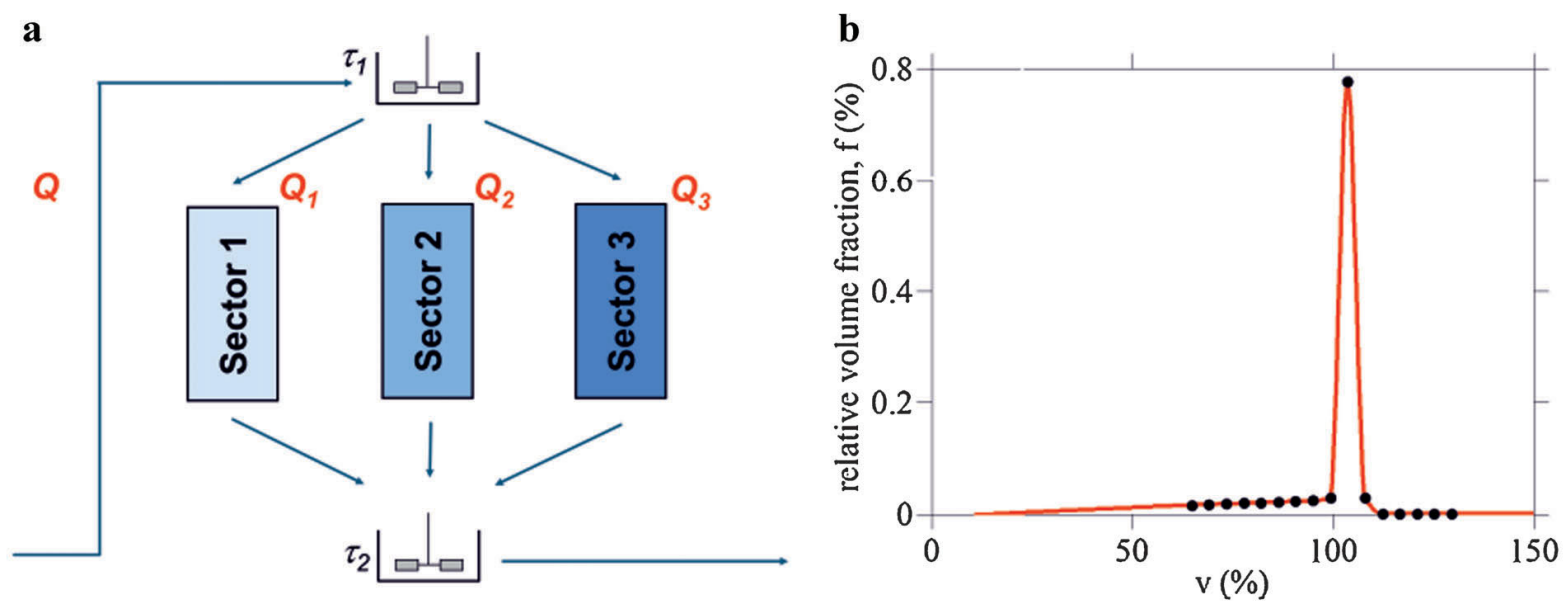
**a**: Virtual partitioning of hold-up volumes and of the membrane for a radial flow configuration in which one axial membrane zone is splitted into three angular sectors with different linear velocities, (**b**): distribution of the volumetric flow relative to the total volumetric flow, *f*, over the linear velocity relative to the average linear velocity, *v*, in the respective sector.

The ZRM quantitatively accounts for non-ideal flow in the void volumes of the XT140 capsule, and the binding parameters are determined independently from the flow regime. Hence, the observed deviations must be caused by capsule specific issues that are negligible under non-binding conditions. An MRI scan reveals that the membrane pleats are not perfectly arranged in the used XT140 capsule. The red arrows in Figure 7b indicate irregular pleats with varying membrane areas. These variations can cause local deviations in the linear velocity that are not accounted by the ZRM, as configured according to Figure 3c. Hence, the data is re-analyzed with a novel configuration of the ZRM in which the axial membrane zone is splitted into several angular sectors with different linear velocities (see Fig. 10a).

The configurations in Figures 3c and Figure 10a can be combined for a ZRM with more than one membrane zone and several sectors with different linear velocities. However, one axial membrane zone has been shown already to accurately describe the XT140 capsule under non-binding conditions (Radial Flow Configuration at Non-Binding Conditions Section), and a second axial membrane zone does not improve the model-based prediction of the XT140 binding data in Figure 9 (data not shown). Varying linear velocities in different sectors of the membrane zone are negligible under non-binding conditions, because the membrane stack is very thin and, consequently, the residence time of the solute molecules in the membrane stack is much shorter as in the hold-up volumes. Nonetheless, varying linear velocities do significantly impact on the loading of the membrane sectors, as the solute molecules are supplied at different rates. The ZRM has two additional parameters for each angular section, the volumetric flow through this section and the linear velocity within this section. However, the overall model has one degree of freedom less when the total volumetric flow rate is given, for example in a three sector model, the relation *Q*_1_ *+* *Q*_2_ *+* *Q*_3_ *=* *Q* allows to compute *Q*_3_ from *Q*_1_ and *Q*_2_.

**Figure 11:**
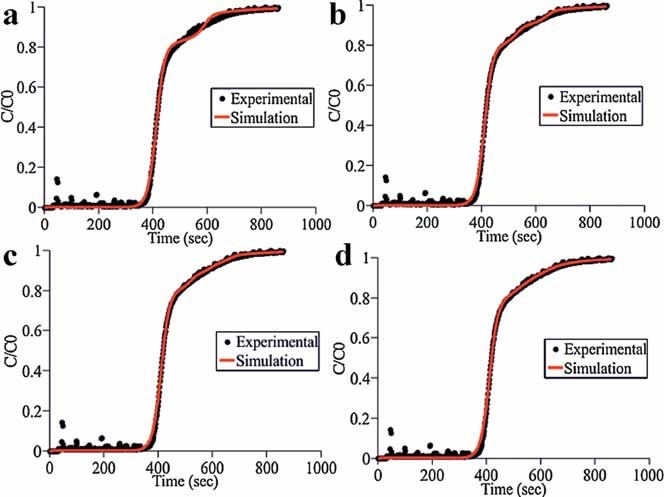
Measured breakthrough curve of the axial flow XT140 capsule under binding conditions. Best fit of the ZRM with one axial membrane zone and (**a**) two, (**b**) three, (**c**) four, and (**d**) sixteen angular sectors.

The measured breakthrough data is first re-analyzed with ZRM configurations with one axial membrane zone and two to four angular sectors (see Fig. 11a–c). Table V shows the fitted volumetric flow through the angular sectors relative to the overall volumetric flow and the linear velocities in these sectors relative to the average linear velocity. Figure 11a–c illustrates that the revised configuration of the ZRM can quantitatively reproduce the measured breakthrough curve. The simulated breakthrough curve increasingly adapts to the measurement data with increasing numbers of sectors. The visible steps in Figure 11a,b are due to the fact, that the ZRM with two and three sectors only coarsely approximates the true velocity distribution. The fitted parameters in Table V reveal that more than 85% of the overall volumetric flow has only a slightly increased linear velocity, whereas the remaining fraction of the volumetric flow has significantly decreased linear velocities. This coincides with the observation that most of the pleats in the used XT140 capsule are quite regular (see Fig. 7b).

**Table V:** Volumetric flow relative to the total volumetric flow and linear velocity relative to the average linear velocity for the asymmetric ZRM with one axial membrane zone and one to four angular membrane sectors as determined by fitting the ZRM to a binding breakthrough curve of the radial flow XT140 capsule

	ZRM with 1 sectors		ZRM with 2 sectors		ZRM with 3 sectors		ZRM with 4 sectors	
				
Sectors	Volumetric velocity (%)	Linear velocity (%)	Volumetric velocity (%)	Linear velocity (%)	Volumetric velocity (%)	Linear velocity (%)	Volumetric velocity (%)	Linear velocity (%)
1	100	100%	88	103	87	103	86	104
2	—	—	12	72	8	83	6.6	82
3	—	—	—	—	5	70	3.5	72
4	—	—	—	—	—	—	3.9	64

The ZRM with four sectors describes the measurement data very well but comprises too many parameters that need to be estimated from experimental data. The following approach is applied for reducing the number of parameters and at the same time to account for the fact, that the true velocity distribution is a continuous function: The ZRM is configured with 16 sectors, and a series of equidistantly spaced linear velocities is assigned to these sectors. The distribution of the total volumetric flow through these sections is approximated by a function that depends on only three parameters (see Fig. 10b). The first two parameters describe the position and the width of the main peak, which is modeled by a Gaussian distribution. The third parameter describes the slope of a linear increase starting at the origin. On the left hand side of the peak, the maximum of these curves is taken. The area under the curve is normalized such as to maintain the total volumetric flow rate. The parameters for the sectors are simultaneously estimated by fitting the ZRM to the measured breakthrough curve.

Figure 11d shows an excellent fit with only three additional parameters. The volumetric flow rate and the linear velocity in the sectors are expressed in relation to their total or average values, respectively. The peak in Figure 10b is slightly shifted to the right, because the average is decreased by the existence of smaller velocities. As before, approximately 85% of the total volumetric flow has almost the same linear velocity, whereas the remaining 15% have significantly different linear velocities.

## Conclusions

The ZRM has previously been applied for analyzing the performance of axial flow membrane chromatography capsules by independently determining the impacts of flow and binding related non-idealities on measured breakthrough curves. In the present study, the ZRM was extended to radial flow configurations and applied for a rigorous analysis of the axial flow XT5 capsule and the radial flow XT140 capsule from Pall. For both capsules, the residence times of the CSTR network were first determined from non-binding data. A symmetric configuration with two membrane zones and four model parameters was required for quantitatively reproducing breakthrough curves of the XT5 capsule, whereas an asymmetric configuration with one membrane zone and three parameters was sufficient for the XT140 capsule. This indicated that the flow is distributed more homogeneously in the XT140 capsule than in the XT5 capsule. Hence, the transfer of binding parameters from one capsule to the other must be accompanied by quantitative modeling of the different flow geometries. Binding data of the XT5 capsule was used for identifying a suitable binding model and determining the corresponding model parameters. The spreading model with six parameters was found to be both physically meaningful and able to reproduce the measurement data much better than the Langmuir model with three parameters. The spreading model is based on the hypothesis of different binding orientations, which might still oversimplify the physical reality of the binding mechanism. However, the model was found to be the best compromise between the number of model parameters and the quality of data fits.

Quantitative reproductions of the individual breakthrough curves in both the simulations and the measurements are essential for a consistent analysis across flow configurations and operating conditions. A revised cleaning protocol with 1 M NaCl instead of 1 N NaOH and the minimization of storage times between the experiments was found to be important for getting reproducible measurement data. A first attempt for model-based scale-up was made by combining the binding related parameters of the XT5 capsule with the flow related parameters of the XT140 capsule. This approach technically allows the prediction of the XT140 performance under binding conditions, as the ZRM makes the binding parameters independent from the flow non-idealities in both capsules. Such predictions can potentially save much money, since the predicted XT140 experiments at binding conditions require significantly more material than the other three experiments together, which are required for calibrating the ZRM namely XT5 experiments at binding and non-binding conditions and XT140 experiments at non-binding conditions.

Unfortunately, the attempt of model-based scale-up from the XT5 capsule to the XT140 capsule was not successful. Irregular pleat structures in the XT140 capsule that can lead to local variations in the linear velocity have been identified as potential cause in an MRI analysis. However, more than 85% of the total volumetric flow was found to be transported with the standard velocity, and only the remaining less than 15% were transported with much lower velocities. These variations in the linear velocity could be described by a distribution with only three parameters. The resulting model can consistently and quantitatively reproduces the studied configurations and operating conditions (compare Figs. 5a and b, 6c, and 11d) with only 16 parameters, that is four parameters per data set. Such an integrative analysis would not have been possible either with the Roper and Lightfoot model for external dispersion or with the Langmuir binding model. The novel results once again highlight the universality and potency of the ZRM.

Future work will be focused on performing similar analyses with capsules of different vendors, in particular analyzing the potential of model-based scale-up, different solute molecules, and different operating conditions that include binding and elution steps.
